# Rothmund–Thomson syndrome type 1 caused by biallelic ANAPC1 gene mutations

**DOI:** 10.1002/ski2.12

**Published:** 2021-02-12

**Authors:** B. Zirn, U. Bernbeck, K. Alt, F. Oeffner, A. Gerhardinger, C. Has

**Affiliations:** ^1^ Genetikum Stuttgart Genetic Counselling and Diagnostics Stuttgart Germany; ^2^ Department of Pediatrics Rems‐Murr‐Klinikum Winnenden Germany; ^3^ Genetikum Neu‐Ulm Genetic Counselling and Diagnostics Neu‐Ulm Germany; ^4^ Department of Dermatology Faculty of Medicine University of Freiburg Freiburg Germany

## Abstract

**Background:**

Rare syndromic skin disorders may represent a diagnostic challenge.

**Aims:**

We report a unique case associating cutaneous manifestations and developmental delay.

**Materials & Methods:**

The affected 14 months old boy had poikiloderma, facial dysmorphism with deep‐set eyes, atrichia, as well as nail dysplasia and non‐descended testes. In addition, his psychomotor development was delayed. Exome sequencing and molecular karyotyping via array‐CGH (oligo‐array, 180k Agilent, design 22060) were performed.

**Results:**

Mutations in RECQL4 (found in patients with RTS2) were first excluded. In the ANAPC1 gene, a novel combination of a recurrent intronic mutation (c.2705‐198C>T) and a deletion of the second ANAPC1 allele was detected, thus confirming the clinical diagnosis of RTS1. The deletion on chromosome 2q13 comprised further genes and spanned 1,7 megabases. Heterozygous deletions in this region are known as 2q13 microdeletion syndrome and are associated with developmental delay, autism and facial dysmorphism.

**Discussion:**

The genetic findings most probably explain both, the RTS1 features and the developmental delay. Genetic diagnosis in RTS is indispensable to confirm the specific subtype and its associated risks: juvenile cataracts are features of RTS1 (ANAPC1 gene), whereas a high risk of osteosarcoma is part of RTS2 (RECQL4 gene). Thus, the patient described here is at high risk for the development of juvenile cataracts and requires regular ophthalmologic examination.

**Conclusion:**

This case report underlines the necessity of thorough clinical diagnosis prior to genetic diagnosis of RTS1, since the recurrent intronic ANAPC1 mutation is otherwise missed.

1

**What is known about this topic?**

- Rothmund–Thomson syndrome type 1 (RTS1) is characterized by poikiloderma, atrichia, facial dysmorphism and juvenile cataracts.
- Causative biallelic mutations in the ANAPC1 gene were found in only 10 patients to date.

**What does this study add?**

- The first patient with an intronic ANAPC1 mutation and a heterozygous chromosome 2q13 deletion, which implies an additional risk for psychomotor delay.
- Specific clinical diagnosis of RTS1 is compulsory; otherwise the recurrent intronic ANAPC1 mutation will be missed in exome analysis.

## 2 INTRODUCTION

Rothmund–Thomson syndrome (RTS) is a rare autosomal–recessive disorder characterized by poikiloderma (hypo‐ and hyperpigmentation, telangiectasias and skin atrophy), sparse hair, eyebrows and eyelashes, short stature, and skeletal anomalies. Two subtypes of RTS are distinguished clinically and genetically. RTS type 2 (RTS2) is caused by biallelic mutations in the RECQL4 gene and is associated with an increased susceptibility to cancer, particularly osteosarcoma.^1^ ^,^ ^2^ RECQL4 belongs to a family of DNA helicases that plays an important role in maintaining genomic stability.^3^

RTS1 is distinct from RTS2 with respect to two clinical aspects: the absence of osteosarcoma and the occurrence of juvenile cataracts in all patients.^4^ The genetic cause of RTS1 has only recently been described in 10 individuals from seven families.^5^ Most interestingly, whole exome sequencing did not reveal biallelic mutations in any coding gene. However, homozygosity mapping displayed a single region of homozygosity in several Amish individuals on chromosome 2q13‐q14.1, and subsequently, expression studies showed decreased levels in only of the included genes, namely ANAPC1. Focused search for noncoding variants of ANAPC1 in the exome data revealed a variant in intron 22 (c.2705‐198C>T) in all 10 RTS1 patients: 5 patients were homozygous for this variant and further 5 patients were compound heterozygous for the intronic mutation and any of three other coding ANAPC1 mutations (c.4882_4883del, c.1778dupA, c.4373+1G>A). All patients had typical dermatologic findings and developed bilateral juvenile cataracts.

## 3 CASE REPORT

The patient was born after 34 + 5 weeks of gestation with a birth weight of 2050 g and underwent anonymous adoption directly after birth. Therefore, further information about pregnancy and family history were not available. The boy developed disseminated erythema within the first weeks and received a topical therapy. When he was first seen in a neuropaediatric department at the age of 4 months, the rash especially affected cheeks and back. The skin was extremely dry and tight. Furthermore, the boy had dysmorphic features, especially deep‐set eyes, missing eyelashes and eyebrows, no hair, hypotelorism, and microgenia. All nails were dysplastic and he had non‐descended testis. Ectodermal dysplasia was clinically suspected; however, exome analysis (clinical exome with target enrichment, TruSightOne Expanded, NextSeq500 Illumina) did not reveal a mutation in associated genes.

**FIGURE 1:**
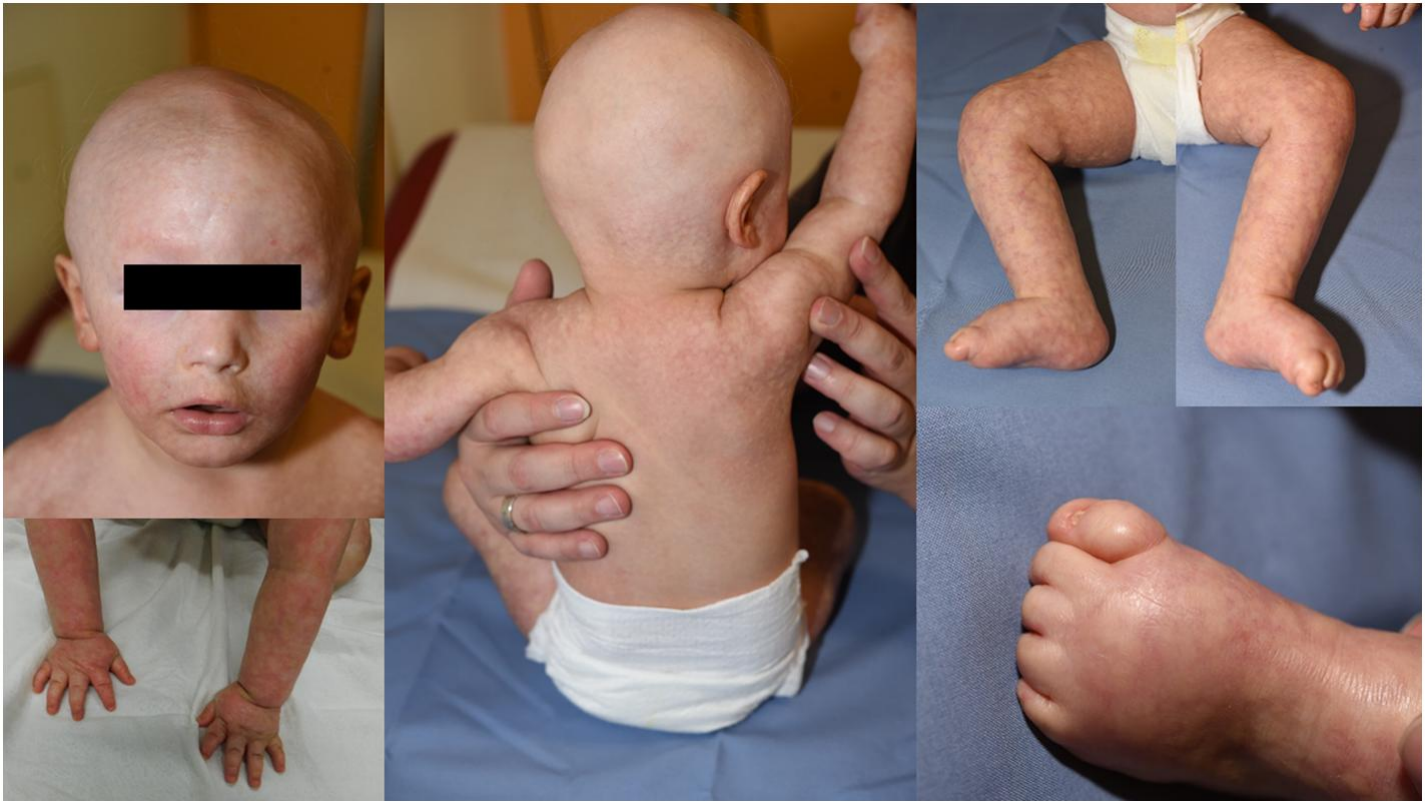
Patient aged 14 months with Rothmund–Thomson syndrome type 1 and typical skin findings: poikiloderma, atrichia, nail dysplasia and progeroid appearance

At the age of 14 months, the child was seen by a specialized dermatologist who diagnosed typical poikiloderma, which had started at the age of approximately 6 months. In addition, atrichia, nail dysplasia and progeroid appearance were noted (Figure 1). Moreover, the boy had growth retardation (length: 3.42 z, weight: 3.08 z), low muscular tone and global developmental delay. Clinically, RTS was suspected. Exome data were reanalysed, and a mutation in the RECQL4 gene (RTS2) was excluded. However, the recently described intronic variant c.2705‐198C>T in the ANAPC1 gene, which happened to be covered in the exome, was detected in heterozygous state and confirmed by Sanger sequencing. Since exome data indicated an additional heterozygous deletion of the ANAPC1 gene, molecular karyotyping via array‐CGH (oligo‐array, 180 k; Agilent, design 22 060) was performed and confirmed a heterozygous deletion on chromosome 2q13, comprising 1.7 Mb and 10 genes (BUB1, ACOXL, BCL2L11, LOC541471, ANAPC1, MERTK, TMEM87B, FBLN7, ZC3H8, ZC3H6).

## 4 DISCUSSION

The ANAPC1 gene encodes the APC1 protein, which is a subunit of the anaphase‐promoting complex/cyclosome (APC/C) and plays an important role in cell cycle progression, apoptosis and senescence. Ajeawung et al.^5^ were the first to describe ANAPC1 mutations in 10 patients with RTS1. All patients presented with typical RTS1 features such as poikiloderma, bilateral juvenile cataracts, as well as abnormal nails and hypotrichia. RECQL4 mutations which cause RTS2 were excluded in all patients. Most interestingly, all patients underwent exome sequencing and were shown to carry a recurrent mutation in intron 22 of the ANAPC1 gene (GenBank: NM_022662.3: c.2705‐198C>T). This mutation was present in five patients in homozygous state. The other five patients had compound heterozygous mutations consisting of the recurrent intronic ANAPC1 mutation c.2705‐198C>T in combination with another exonic mutation. Functionally, it could be demonstrated that the recurrent intronic ANAPC1 mutation c.2705‐198C>T causes a pseudoexon which triggers nonsense‐mediated decay and leads to decreased ANAPC1 protein levels in humans.

The boy depicted here has a unique combination of the recurrent intronic ANAPC1 mutation with a microdeletion of chromosome 2q13, including the ANAPC1 gene. Heterozygous microdeletions in 2q13 (same size of 1,7 megabases and 10 included genes) were independently described in patients with unspecific variable intellectual disability and attention disorders as well as craniofacial abnormalities.^6^ Thus, the boy's genetic diagnosis explains both the RTS phenotype and the developmental delay. He is at high risk for bilateral juvenile cataracts and needs regular ophthalmological examinations.

The boy's biological parents are obligate carriers of one of the heterozygous ANAPC1 mutations and have 25% recurrence risk of RTS1 with biallelic mutations in further children. Moreover, the parent with heterozygous microdeletion 2q13, including ANAPC1 has a 50% transmission probability with elevated risk of developmental delay and intellectual disability in person and in further children. Due to the anonymous adoption of the child, this information could not be rendered to the biological parents.

In summary, genetic diagnosis is mandatory to assess the specific prognosis in the two subtypes of RTS: the high risk of bilateral juvenile cataract in RTS1 (ANAPC1) and the cancer risk, especially for osteosarcoma in RTS2 (RECQL4). ANAPC1 mutations are missed in routine exome analysis since the typical intronic mutation is either not covered or not analysed. Our case demonstrates that primary clinical syndrome diagnosis is essential to genetic diagnosis and that dermatologic key manifestations such as poikiloderma like in this case can guide the genetic diagnosis.

## CONFLICT OF INTERESTS

The authors declare that there is no conflict of interests.

## References

[1] Kitao S , Shimamoto A , Goto M , et al. Mutations in RECQL4 cause a subset of cases of Rothmund‐Thomson syndrome. Nat Genet. 1999;22:82–84. 10319867 10.1038/8788

[2] Lu L , Jin W , Wang LL . RECQ DNA helicates and osteosarcoma. Adv Exp Med Biol. 2020;1258:37–54. 32767233 10.1007/978-3-030-43085-6_3

[3] Yokoyama H , Moreno‐Andres D , Astrinidis SA , et al. Chromosome alignment maintenance requires the MAP RECQL4, mutated in the Rothmund‐Thomson syndrome. Life Sci Alliance. 2019;2: e201800120. 30718377 10.26508/lsa.201800120 PMC6362308

[4] Wang LL , Plon SE . Rothmund‐Thomson syndrome In: MargaretPA, HollyHA, RobertaAP, StephanieEW, LoraJHB, KarenS, AnneA, editors. GeneReviews® [Internet]. Seattle, WA: University of Washington; 1999 pp. 1993–2020.

[5] Ajeawung NF , Nguyen TTM , Lu L , et al. Mutations in ANAPC1, encoding a scaffold subunit of the anaphase‐promoting complex, cause Rothmund‐Thomson syndrome type 1. Am J Hum Genet. 2019;105:625–30. 31303264 10.1016/j.ajhg.2019.06.011 PMC6731352

[6] Hladilkova E , Barøy T , Fannemel M , et al. A recurrent deletion on chromosome 2q13 is associated with developmental delay and mild facial dysmorphisms. Mol Cytogenet. 2015;8:57. 26236398 10.1186/s13039-015-0157-0 PMC4521466

